# Black women’s decisions about participating in breast cancer screening in the UK: a qualitative study

**DOI:** 10.1038/s41416-026-03551-6

**Published:** 2026-07-16

**Authors:** Anietie E. Aliu, Robert Stephen Kerrison, Afrodita Marcu

**Affiliations:** School of Health Sciences, University of Surrey, Surrey, UK

**Keywords:** Cancer screening, Breast cancer, Breast cancer

## Abstract

**Background:**

In the UK, women of Black African and Black Caribbean descent (‘Black women’) participate in breast screening at lower rates than White women, contributing to inequalities in breast cancer outcomes. This study explored Black women’s decision about participating in breast cancer screening.

**Methods:**

Seven focus groups were conducted with 47 women eligible for breast screening; 30 reported consistent participation, while 17 had never or inconsistently participated. Data were analysed using Reflexive Thematic Analysis and interpreted through the Capability, Opportunity, Motivation–Behaviour (COM-B) model. Findings were mapped onto the Behaviour Change Wheel (BCW) to identify potential intervention functions.

**Results:**

Five themes describing Black women’s decisions to participate in breast screening were developed: knowledge and awareness (psychological capability); emotional and motivational responses (reflective and automatic motivation); social influences and peer engagement (social opportunity); competing priorities (psychological capability and physical opportunity); and healthcare and access barriers (physical opportunity). A sixth theme captured participants’ suggestions for improving screening services.

**Conclusion:**

Applying the Behaviour Change Wheel (BCW) identified relevant intervention functions and policy approaches, suggesting that community-driven and culturally inclusive strategies may enhance breast screening participation among Black women.

## Introduction

Breast cancer is the most frequently diagnosed cancer among women globally [1]. Each year, the malignancy accounts for over 2.3 million new cases and 670,000 deaths [1, 2].

In the UK, specifically, 56,800 men and women are diagnosed with breast cancer each year, making it the single most common cancer in England, Scotland, Wales and Northern Ireland [3, 4]. Of those diagnosed in the UK, 11,500 do not survive five or more years, making it the fourth most common cause of cancer-related death [4, 5].

Five-year survival is strongly associated with stage at diagnosis. For example, the five-year survival for stage I breast cancer is >99%, while the five-year survival for stage IV breast cancer is only 25% [3, 5]. Early detection is most likely when a patient participates in mammographic breast screening [6]. As a result, many countries across the world provide population-based screening programmes for the early detection of breast cancer (including many countries within Europe, the Americas, Asia, and Oceania) [7].

In the UK, the National Health Service (NHS) provides population-based mammographic breast screening to women aged 50–71, once every three years [6]. However, despite being free at the point of use, uptake of breast cancer screening is suboptimal, and there are stark inequalities in participation between demographic groups. For example, women living in deprived neighbourhoods, women with severe mental illness, and women from ethnic minority groups are significantly less likely to participate in breast cancer screening, compared with their respective counterparts [8].

Women of Black African and Black Caribbean descent (hereafter referred to as ‘Black women’,) have the lowest uptake of any ethnic group in the UK [9], with similar trends among African American women in the USA [10]. Indeed, in a recent study, Kerrison et al found that between March 2016 and September 2020, only 45% of Black women participated in breast screening compared to 63.0% of White women [8].

These differences in breast screening uptake are thought to contribute to observed inequalities in stage at diagnosis and overall survival: 25% of Black African and 22% of Black Caribbean women are diagnosed at stage III/IV, compared to only 13% of White women [11].

To date, there have been few studies exploring the reasons why Black women, living in the UK, are less likely to participate in breast cancer screening [12]. Those that have been conducted have primarily involved Black women who either participate in breast screening or are not yet eligible to participate in breast screening [12]. The views of non-attenders, specifically, have not been explored. Further, previous research has conceptualised Black women as a homogenous group, overlooking important potential differences in terms of religious beliefs (e.g. Christian vs Muslim), ethnicities/nationalities (e.g. Black African vs Black Caribbean) and generational differences (i.e. born within the UK vs outside of the UK) [12]. The current study aims to address these gaps by including screening eligible Black women who do not attend breast screening, comparing the views of Black African and Black Caribbean women, and reflecting on subgroup differences, including place of birth and religious beliefs.

## Methods

### Study design and theoretical framework

This qualitative study used semi-structured focus groups to explore how Black women in the UK engage with breast cancer screening. A qualitative approach enabled in-depth exploration of participants lived experiences, beliefs, and decision-making processes.

The study was informed by the Capability, Opportunity, Motivation-Behaviour (COM-B) model, which provides a structured framework for understanding the behavioural drivers of health-related actions [13, 14]. The COM-B model conceptualises behaviour as the interaction between capability (knowledge and skills), opportunity (social and environmental factors), and motivation (reflective and automatic processes) [14]. To enhance the applied relevance, findings were subsequently mapped onto the Behaviour Change Wheel (BCW), which links behavioural determinants to intervention functions and policy strategies [14, 15].

A semi-structured topic guide (Appendix 1) was developed based on the COM-B model and existing literature on barriers to cancer screening [9, 12]. Questions explored knowledge and understanding of breast cancer and screening (capability), structural and social influences affecting access (opportunity), and emotional responses, beliefs, and motivations related to screening (motivation). The guide was iteratively refined with input from patient and public involvement (PPI) representatives to ensure clarity, cultural relevance, and appropriateness for the target population.

### Sampling and recruitment

Participants were recruited using purposive sampling to ensure inclusion of women with diverse screening experiences, including those who had attended screening regularly, those who had attended inconsistently, and those who had never attended screening despite receiving invitations. Eligible participants were Black African or Black Caribbean women aged 50–71 years, corresponding to the age range when women are invited for routine breast screening within the NHS Breast Screening Programme [6]. Women with a previous breast cancer diagnosis were excluded.

Screening history was self-reported and used to categorise participants into three groups: consistent attenders (attended screening whenever invited), inconsistent attenders (attended at least once but missed one or more invitations), and consistent non-attenders (never attended despite receiving invitations). This categorisation enabled the understanding of women’s decision-making and motivations to attend screening or not.

Recruitment was conducted through social media platforms (e.g., X, Facebook, LinkedIn), patient and public involvement networks, and community organisations, with an emphasis on engaging women from areas with lower screening uptake, e.g. London [16]. Efforts were made to recruit participants from diverse cultural and religious backgrounds to capture a range of experiences.

Sample size was guided by the concept of information power, whereby adequacy is determined by the richness and relevance of the data rather than numerical size. Studies with a focused aim, strong theoretical underpinning, and rich data require fewer participants to achieve sufficient analytical depth [17]. Given the focused study aim, use of established theoretical frameworks (COM-B), and depth of data generated through focus groups, the sample was considered sufficient to address the research question.

### Data collection

Seven focus groups and one individual interview were conducted between August 2024 and January 2025. Focus groups were selected to facilitate interaction and collective discussion, allowing participants to reflect on and respond to each other’s experiences and thereby reveal shared norms and social influences [18, 19]. One participant who was unable to attend a focus group participated in an individual interview. The size of each of the focus groups varied from four (*n* = 4) to eight (*n* = 8). Sessions were moderated by the lead researcher (AA) using the semi-structured topic guide. Discussions lasted between 60 and 90 min and were conducted in community settings. An online session was however organised for Black women outside of London.

All sessions were audio-recorded with participants’ consent and transcribed verbatim. Field notes were taken during data collection to capture contextual observations and support interpretation during analysis.

### Data analysis

Data were analysed using reflexive thematic analysis, following the approach outlined by Braun and Clarke [20–22]. Transcripts were managed and coded in NVivo 14 [23] to support organisation of codes and theme development. Analysis followed six iterative phases: familiarisation with the data, generation of initial codes, development of candidate themes, refinement of themes, definition and naming of themes, and production of the final analytic narrative [22, 24]. An inductive approach was initially used to ensure that themes were grounded in participants’ accounts. This was followed by a deductive stage in which themes were mapped onto the COM-B model to organise findings according to behavioural domains.

To enhance analytical rigour, coding and theme development were discussed iteratively within the research team (AM and RK), ensuring reflexive engagement with the data and consistency in interpretation [25]. COM-B was applied to structure understanding of behavioural drivers, while the Behaviour Change Wheel (BCW) was used to identify potential intervention functions and inform future strategies and policies [13, 14].

### Reflexivity

Reflexive thematic analysis recognises that knowledge is co-constructed between researcher and participants [21]. The lead researcher (AA), a Black British woman with a clinical background in nursing, brought both cultural and professional insight to the research. This positionality facilitated rapport with participants and a nuanced understanding of the healthcare contexts discussed.

At the same time, reflexive practices were employed to critically consider how these perspectives may shape data interpretation. Ongoing reflection and discussion within the research team supported transparency and reflexivity throughout the analytic process [18, 19].

### Patient and public involvement

Patient and public involvement (PPI) was integral to the study design. Black community advisors and representatives from relevant organisations contributed to the development of study materials, including the topic guide and recruitment strategies.

Two online sessions were held with a PPI cancer research group comprising individuals from diverse backgrounds to refine the research focus and ensure cultural appropriateness of the study approach.

## Results

### Participant characteristics

Between August 2024 and January 2025, seven focus groups and one individual interview were conducted with Black women aged 50–71 years (*N* = 47). Participants self-identified as Black African (*n* = 28, 59.6%) or Black Caribbean (*n* = 19, 40.4%), with most born outside the UK (*n* = 29, 61.7%). Nearly two-thirds reported consistent participation in breast screening (*n* = 30, 63.8%), while others reported inconsistent (*n* = 2, 4.3%) or no participation (*n* = 16, 31.9%). Most had at least post-secondary education (*n* = 20, 42.6%), and about a third were in employment (*n* = 22, 36.7%).

Participants were purposively grouped to reflect shared cultural or religious backgrounds where appropriate, facilitating open discussion of sensitive issues, for instance, there were separate focus groups with Caribbean women, Muslim women and Christian women and some were mixed. A summary of participant characteristics is presented in Table 1.

**Table 1:** Sample characteristics.

Characteristic	*N* (%)
**Age (years)**	
250–60	25 (53.2)
61–71	22 (46.8)
**Ethnicity**	
Black African	28 (59.6)
Black Caribbean	19 (40.4)
**Place of birth**	
UK	18 (38.3)
Other	29 (61.7)
**Marital status**	
Married	17 (36.2)
Not married	26 (55.3)
Missing	4 (8.5)
**Educational attainment**	
Degree or higher	16 (34.0)
A-levels or lower	20 (42.6)
Missing	11 (23.4)
**Employment status**	
Employed full time	9 (19.1)
Employed part time	9 (19.1)
Self-employed	4 (8.5)
Retired	7 (14.9)
Unemployed/homemaker	8 (17.0)
Missing	10 (21.4%)
**Religion**	
Christian	32 (68.1)
Islam	10 (21.3)
None	5 (10.6)
**Screening history**	
Consistent attenders	30 (63.8)
Inconsistent attenders	2 (4.3)
Consistent non-attenders	15 (31.9)

#### Key themes

The prefix on participants pseudonymised names signifies participants’ ethnicity, the focus group/interview and the screening participation status (for example: BA -Black African, BC -Black Caribbean, FG- focus group’ IN—interview, A-attender, IA—inconsistent attender and NA -non-attender).

Five overarching themes were developed and mapped onto the Capability, Opportunity, Motivation–Behaviour (COM-B) model: (1) knowledge and awareness of breast cancer and screening (psychological capability); (2) emotional and motivational responses (reflective and automatic motivation); (3) social influences and peer engagement (social opportunity); (4) competing priorities (psychological capability and physical opportunity); and (5) healthcare and access barriers (physical opportunity). A sixth theme captures participants’ suggestions for improving screening services. The mapping of these findings to COM-B constructs and relevant Behaviour Change Wheel intervention functions is summarised in Table 2.

**Table 2:** Mapping of findings to COM-B constructs and BCW functions.

COM-B construct	Aspects	Barriers (findings)	Facilitators (findings)	Relevant BCW intervention functions
**Capability**	*Psychological capability* (knowledge, understanding); *Physical capability* (skills)	Limited awareness of breast cancer and screening; Misinformation and myths; Low perceived risk	Clear, accessible screening information; Knowledge from family/friend, cancer experiences	**Education** (increase knowledge) **Training** (practical guidance) **Enablement** (tailored information, myth-busting)
**Opportunity**	*Physical opportunity* (environment, resources) *Social opportunity* (norms, cultural influences)	Inflexible appointment systems; Difficulties accessing services; Past negative healthcare experiences; Cultural and religious norms discouraging screening	Flexible, community-based services; Accessible local screening centres; Supportive cultural/religious messaging	**Environmental restructuring** (improve service accessibility) **Enablement** (reduce logistical barriers) **Modelling** (community and faith leaders promoting screening), community champions, promote trust
**Motivation**	*Reflective motivation* (beliefs, intentions) *Automatic motivation* (emotions, habits)	Fear of pain or diagnosis; Fatalistic beliefs about cancer; Competing priorities (family/work); Low trust in healthcare	Peace of mind and reassurance from early detection; Positive prior experiences; Peer/family encouragement	**Persuasion** (highlight benefits, address fear) **Incentivisation** (emphasise reassurance and early detection) **Modelling** (survivors testimonials, peer encouragement)

##### Knowledge and awareness of breast cancer and screening (Psychological capability)

Awareness of breast screening varied considerably, particularly by place of birth. Many women born outside the UK reported limited prior knowledge of screening programmes before receiving their first NHS invitation:

*“I just knew about it when I was 50… I didn’t know anything until I got the letter” (BAJ2, FG2, A)*.

*“…if you’ve just come 10 years ago, in your country you don’t have it, it won’t click in your mind” (BAMB2, FG3, NA)*.

In contrast, UK-born participants, particularly Black Caribbean women, described greater familiarity with the healthcare system and screening processes: “As a second generation… we understand the NHS screening.” (BCB, FG6, A). However, the understanding of screening purpose differed. Attenders frequently described screening as a preventive and life-saving measure: *“I see it as a way of prevention and lifesaving” (BCT2, FG7,A)*, whereas non-attenders often questioned its relevance in the absence of symptoms, demonstrating low awareness:

*“My breasts are healthy… I don’t see anything… why should I go?” (BAM2, FG1, NA)*.

*“Why am I going when I don’t have any problem? “So, I thought, maybe I’m okay. But if I see any alarm, I’m going to go there” (BAMX, FG3, NA)”*

Some participants reported relying on self-examination or previous normal results as a substitute for regular screening, reflecting gaps in understanding of the need for routine participation:

*“I know how I can check myself; I attended a training…so I say, I don’t need to go into that machine” (BAJ2, FG2, NA)*.

*“Because I was cleared over 10 years ago, so I don’t want to live in fear…. I don’t feel nothing” (BAM2, FG1, NA)*.

These findings reflect gaps in psychological capability, particularly in knowledge, risk perception, and understanding of preventive screening.

##### Emotional and motivational responses (Motivation)

Emotional responses played a central role in shaping screening behaviour. Fear of cancer diagnosis was reported by Black women across all focus groups, reflecting widespread anxiety about receiving a cancer diagnosis. However, fear of anticipated pain was more commonly reported among non-attenders and contributed to reluctance to participate in screening. Concerns about body image and the potential impact of treatment, particularly mastectomy, also influenced emotional responses, with some women expressing anxiety about how breast cancer might affect their identity and femininity.

*“I can imagine the big C word is very scary… you think about having your breast taken off” (BAG2, FG2, NA)*.

*“My aunt said it was quite painful… I don’t want pain, so I did not go” (BAMX, FG3, NA)*.

*“People are afraid about that because, what happened, you’re nice now, but if you don’t have no boobs, how are you going to be recognised? It’s kind of mental” (BAMA2, FG3, NA)*.

Anxiety was also expressed in relation to screening environments. Some women avoided hospital-based appointments due to fear of infection: *“I don’t even want to be in that hospital setting; you go in there and come out with infections… I would have liked the portacabin {community mobile units}, it would have made me less scared.” (BCU2, FG7, NA)*. Others were concerned about the test outcomes and the perceived effects of mammogram X-rays: *“You know X-rays, machines they kill some of our cells, yeah, that’s why I did not go” (BAG2, FG2, NA)*. Some non-attenders were also anxious about the screening procedure and who would perform the test, reflecting potential trust issues: *“For me, the person I meet is very important… who will be checking me, will someone spoil my day again, will they be nice to me? and how are they going to check me?” (BAMX, FG3, NA)*.

Concerns about the accuracy of screening results further contributed to anxiety and uncertainty. Some participants questioned whether mammography could reliably detect cancer or feared false-positive or missed diagnoses. For example, one participant stated, *“Thinking of the mammogram results, there’s been incidents in the past where people end up with result of positive breast cancer that was not true” (BCBE, FG6, NA)*. Another reflected uncertainty about test reliability: *“But people are carrying out the tests, not having that accuracy… because you are going, test, test, test, and then suddenly, they say, oh, you’ve got stage four. So, I’m thinking, so what were they checking all along?” (BCCH, FG6, A)*.

Fatalistic beliefs were also evident, with cancer often associated with death *“When I hear the words cancer, ultimately there’s a sense of death that’s associated with it” (BCR2, FG7,A)*. Importantly, cancer was sometimes described indirectly or avoided altogether in conversation, reflecting cultural silence and the emotional weight associated with the disease:

*“The word ‘cancer’ first brings to mind death… with fear” (BAN2, FG1, A)*.

*“We don’t mention the name {cancer}, we just say the big letter ‘C’” (BAL, FG5, NA)*.

At the same time, screening was perceived by some as reassuring, particularly among attenders and those with prior exposure to cancer:

*“If you have direct first-hand family experience… certainly yes [you go]” (BCO2, FG7, A)*.

*“That’s why I did mine as well. Yeah… after I heard about my sister {breast cancer diagnosis}, I go and I ask my doctor, please can I have one?” (BAMW,FG3,A)*

For several non-attenders, low motivation was not always linked to lack of awareness but rather to postponement or low prioritisation:

*“Nothing was on my mind… I was just stalling” (BCE, FG6, NA)*.

Faith and religious beliefs influenced decision-making in varied ways. For some, faith provided reassurance and supported engagement with screening, while for others it contributed to perceptions of invulnerability or reduced perceived need. For instance, some Black African Christians maintained that illness including cancer was not permitted by God in their bodies. *“I am a believer… no sickness is permitted in my body including cancer” (BAM2, FG1, NA)*, while others saw screening as a personal choice that does not conflict with Christian faith. For instance, a Christian Caribbean woman saw it as a *“blessing from God”* (BCG2, FG7, A) and a Muslim African participant said it was using the knowledge Allah gives them: *“So, using that knowledge to do something good for your health” (BAMW, FG3, A)*. However, most Caribbean women said religion did not strongly influence their health decisions, in contrast to Muslim women who emphasised that indicating their religion in the medical appointment form was important to guarantee a female mammographer: *“When you book an appointment, you have to put your religion, so they make sure it’s a woman” (BAC2, FG2, A)*.

These findings portrays both reflective motivation (beliefs about risk and outcomes) and automatic motivation (emotional responses such as fear and anxiety).

##### Social influences and peer engagement (Social opportunity)

Social interactions emerged as an important influence on screening decisions. Within focus group discussions, attenders often challenged misconceptions and encouraged others to reconsider non-attendance:

*“Excuse me, that is wrong, people should apply their knowledge” (BAK2, FG1, A)*.

*“There forgot the risk, I think it is lack of knowledge, we don’t talk about [breast screening] enough.” (BCN, FG4, A)*.

In addition, a Black African women tried to debunk the pain myths: *“What I wanted to contribute, ladies, does this pain stay for the whole day? And if you compare it, …like when you’re in labour, there is no comparison” (BAV, FG3,A)*.

These exchanges appeared to influence attitudes, with some non-attenders reporting increased motivation following discussion:

*“…now that I’ve had this discussion… it’s motivated me to begin to do my breast screening” (BACI, IN, NA)*.

*“I was invited twice; I just ignored it. But after this I will call and book for my screening” (BAI, FG5,NA)*.

*“… I was told it was quite painful, from what you have all said, it may not be that bad, I will go and try” (BAMX, FG3, NA)*.

These interactions suggest that social opportunity, including peer influence and community dialogue, may play a role in shaping screening perceptions, attitudes and behaviours to promote engagement with screening.

#### Competing priorities (capability)

Participants described how competing life demands often constrained their ability to attend screening. Work responsibilities, financial pressures, and caregiving roles frequently took precedence over preventive health behaviours.

*“I have two jobs; I don’t have the time… If there was pain, I would sacrifice time” (BAI,FG5,NA)*.

*“As an ethnic minority you want to work, you need to earn… you prioritise those things over… your body” (BACI, IN, NA)*,

Limited flexibility within employment settings further restricted attendance, particularly where women felt unable to negotiate time off or reschedule appointments. Screening was therefore often deprioritised unless symptoms were present. Some women felt they had to *“ask if we can go”, and “if your line manager says no because it’s not convenient, then we don’t rearrange*, *appointments got missed*” (*BCN, FG4, A)*. This highlights constraints in both psychological capability (prioritisation of health) and physical opportunity (time and access to health services).

##### Healthcare and access barriers (Physical opportunity)

Experiences within healthcare systems influenced trust and engagement with screening services. Some participants described feeling dismissed or not listened to, which contributed to reluctance to seek care. For example, one participant reported, *“The GP brushed off my pain… so I fear future misdiagnosis” (BACI, IN, NA)*. Others highlighted how healthcare professionals’ attitudes affected their comfort and willingness to engage, noting that *“it depends on the individual that’s attending to you… some of them, the body language puts you off” (BAK, FG5, A)*. Participants also raised concerns about cultural competence, including limited awareness of how symptoms may present on darker skin.

Communication during screening procedures emerged as an important factor shaping experiences. Some participants described insufficient explanation of the procedure and unclear communication from healthcare professionals:

*“I was just told to go and stand by machine, turn right, turn left, the next thing, boom, I’m feeling my breath and then I screamed… if someone had explained… more like someone preparing your mind with what’s coming…” (BAFA, FG3, IA)*.

In contrast, clear communication was identified as a facilitator, with participants reporting that explanations reduced anxiety and improved experiences: *“If they explain what they’re going to do… it makes you feel more at ease” (BAQ, FG4, A)*.

Practical access barriers were also reported, including inflexible appointments and limited continuity of care. Proximity and convenience of screening locations influenced attendance, with community-based or nearby services viewed positively. Participants stated:

***“****…but I would have more flexibility to attend somewhere more convenient to access” (BCT, FG4, NA)*.

*“But it’s better they try to… be near to the community where people live” (BAMSI, FG3, NA)*.

While another described difficulties travelling to appointments:

*“…it would be nice to be near… where I went, it was 2 miles. I was even late. I missed my way, I was confused” (BAMET, FG3, IA)*.

These findings highlight limitations in physical opportunity, including access to services, quality of interactions, and challenges navigating the healthcare system.

##### Suggestions for improvement

Participants proposed several strategies to improve screening uptake. These included clearer, more accessible communication, use of visual or multimedia information, and reminders beyond written letters:

*“We do not read long letters… women like to listen or watch something” (BAK, FG5, A)*.

Flexibility in appointment systems, including self-booking and evening or weekend options, was strongly recommended. Participants also supported the use of community-based screening services and mobile units in familiar settings:

*“If I could just do my shopping, pop into the Portacabin and get it done… it normalises it” (BCS2, FG7, NA)*.

*“… but I would have more flexibility if I could actually self-select…” (BCO, FG4, IA)*.

The participants also suggested collaboration between the primary care and Black community champions to facilitate screening awareness. The GPs and patients teaming up together would promote trust with service users as well as enable health and screening representative information to be communicated.

*“Most surgeries have an empty room and spaces … why can’t you just have the GP surgeries set one room aside and once a month, community champions will be there to share valuable awareness information with women” (BCF, FG6, A)*.

Culturally tailored awareness campaigns, including representation of Black women and involvement of community leaders or champions, were seen as essential for improving trust and engagement. These recommendations align with BCW intervention functions, including education, environmental restructuring, enablement, and modelling.

## Discussion

### Summary of main findings

This study provides insight into how behavioural, structural, and socio-cultural factors interact to shape Black women’s participation in breast cancer screening in the UK. Interpreting the findings through the COM-B model highlights that screening decisions are influenced by interrelated factors operating across capability, opportunity, and motivation, rather than by single barriers. These findings underlines the complexity of screening engagement and emphasise the need for multi-level strategies to support participation.

Within the capability domain, limited awareness of breast screening, particularly among women born outside the UK, emerged as a key barrier. Screening was often perceived as unnecessary in the absence of symptoms, reflecting low perceived risk and a tendency towards reactive rather than preventive healthcare. Women with prior exposure to cancer through family or social networks demonstrated greater awareness and engagement. The findings also highlight that Black women are not a homogeneous group; differences in place of birth, faith, and lived experiences influenced awareness and engagement.

Black Caribbean women and Black African women differed in their awareness and knowledge of breast screening. The Black Caribbean women reported greater familiarity with the NHS screening programme, mostly because they were UK- born or long-term residents.

Opportunity-related factors also influenced participation. Structural barriers, including inflexible appointment systems, travel distance, and competing work and family demands, constrained women’s ability to attend screening. Experiences within healthcare settings also shaped engagement, with clear communication and respectful care facilitating participation, while perceived dismissal, lack of cultural sensitivity, or poor communication contributed to mistrust and disengagement.

Motivational factors included fear of cancer diagnosis, anxiety about the screening expectations and the attitudes of the healthcare professional doing the test, anticipated pain, and fatalistic beliefs about cancer outcomes, all of which discouraged screening for some women. Conversely, reassurance regarding early detection, encouragement from family and peers, and positive prior experiences acted as strong facilitators. Social dynamics also played an important role; with peer discussions challenging misconceptions and, in some cases, prompted non-attenders to reconsider their decisions.

A key contribution of this study is its focus on women eligible for breast screening, enabling examination of real-world screening decisions rather than hypothetical intentions. By integrating behavioural science frameworks (COM-B and the Behaviour Change Wheel) with qualitative insights, this study provides a structured, theory-informed basis for intervention design and highlights the importance of intersectional differences shaping screening behaviours.

### Comparison with existing literature

The findings of this study are broadly consistent with previous research identifying limited awareness, fatalistic beliefs, and structural barriers as key determinants of breast cancer screening participation among ethnic minority women in the UK [9, 26, 27]. Previous qualitative and mixed-methods studies have also reported that low health literacy, fear of cancer, and competing life priorities contribute to reduced screening uptake among women from Black and other minority ethnic backgrounds [28–30]. Our findings extend this literature by highlighting how fear of cancer diagnosis is shaped not only by fatalistic beliefs but also by perceptions of poor breast cancer outcomes within the Black community, concerns about the debilitating nature of the disease, potential loss of income, and body image issues such as mastectomy. Anxiety about the screening procedure and uncertainty about who would perform the test further influenced women’s decisions.

This study extends the existing literature in several ways. First, by focusing exclusively on women who are eligible for breast screening, this study provides insight into actual screening decisions rather than anticipated, which have been the focus of some previous research [12], strengthening the ecological validity of the findings. Second, the results highlight important within-group differences among Black women, supporting calls for intersectional approaches to understanding screening behaviours [31]. Consistent with previous research, women born outside the UK, particularly Black African women, reported lower awareness of organised screening programmes compared with UK-born women [32, 33]. Our findings further illustrate how place of birth influences familiarity with the healthcare system, cultural beliefs, and prior exposure to preventive care, shaping their engagement with screening.

In addition, while previous studies have largely focused on individual-level barriers, our findings emphasise the role of social dynamics in shaping screening decisions. Participants shared experiences, challenged misconceptions, and encouraged reconsideration by non-attendance within group discussions. This aligns with emerging evidence that social networks and community engagement influence health behaviours [29, 34] and demonstrates how peer dialogue may shift intentions in real time.

Finally, this study contributes to the literature on culturally sensitive healthcare by identifying actionable components of effective communication. Although previous research recommends culturally tailored interventions [29, 35] participants emphasised respectful interactions, active listening and clear explanations of screening procedures. These findings provide practical guidance for improving patient–provider interactions and strengthening trust, which is critical for engagement with healthcare services among underserved populations [36].

### Implications for future research and practice

Applying the COM-B model to the findings provides a structured basis for developing interventions to improve screening participation. Interventions targeting capability should focus on increasing awareness and understanding of breast screening, particularly among women with limited prior exposure to organised screening programmes. This may include culturally tailored education delivered through community settings and multimedia formats. Providing clear information about breast cancer treatment, including modern advances and less invasive options, may also help reduce fear of mastectomy and improve motivation to attend screening.

To address opportunity-related barriers, healthcare systems should increase flexibility in screening provision, including self-booking options, extended hours, and community-based or mobile screening services in familiar environments, such as community centres or faith-based settings. Motivational barriers may be addressed through interventions that reduce fear, anxiety and fatalism while emphasising the benefits of early detection [28]. Incorporating survivor testimonials, peer-led discussions, and community champions may help shift perceptions and increase motivation.

The findings also highlight the importance of culturally responsive care. Healthcare professionals should prioritise respectful communication, active listening, and clear explanations of screening procedures. Tailoring communication to reflect cultural and linguistic diversity may help build trust and improve engagement. Importantly, interventions should avoid treating Black women as a homogeneous group. Instead account for differences in place of birth, cultural background, and faith to ensure that interventions are appropriately tailored to diverse subgroups.

### Strengths and limitations

This study has several strengths. The use of multidimensional approach in exploring barriers across place of birth, religion, ethnicity, and screening history, provides a strategic understanding of subgroup experiences. Importantly, the study focused specifically on screening-eligible women, enabling direct examination of real screening decisions rather than anticipated barriers among women who are not yet eligible for screening. The use of behavioural frameworks (COM-B and BCW) strengthened the analytical approach and facilitated translation of findings into theory-based recommendations for intervention development. In addition, patient and public involvement ensured that the research was culturally relevant and grounded in community perspectives.

However, limitations should be acknowledged. As a qualitative study, findings are not statistically generalisable. Most participants were based in London, which may limit transferability to other regions. Additionally, the exclusion of non-English speakers means that language-specific barriers may not have been fully captured.

## Conclusion

Black African and Caribbean women in the UK face intersecting cultural, structural, and informational barriers to breast screening. Addressing these requires multi-level, co-designed interventions that enhance capability, opportunity, and motivation, such as culturally tailored education, flexible booking, mobile screening, and community-led engagement. Embedding behavioural frameworks within intervention design and amplifying service users and community voices are key to improving early detection and equity in breast cancer outcomes.

## Supplementary information

Semi-structured Focus Group Topic Guide -Appendix 1

## Data Availability

All data files are available via the research team on request.
