# Supplementary material for: Black women’s decisions about participating in breast cancer screening in the UK: a qualitative study
Source: Br J Cancer. 2026 Jul 16;135(8):1232–9. doi: 10.1038/s41416-026-03551-6 (PMC13642912; doi:10.1038/s41416-026-03551-6)
Supplement: Supplementary file 1 [file 41416_2026_3551_MOESM1_ESM.docx]

**Appendix 1. Focus Group Topic Guide (COM-B informed)**

**Introduction**

- Welcome and introductions
- Brief overview of study purpose
- Ground rules (confidentiality, voluntary participation, right to withdraw)
- Ice-breaker question

Participants were informed that the discussion would explore experiences and views about breast cancer screening and how services could be improved for Black African and Black Caribbean women in the UK.

**Domain 1: Capability — Knowledge and Awareness**

- What are your general thoughts about the NHS and health checks offered after age 50?
- What comes to mind when you think about cancer?
- What thoughts or feelings does breast cancer evoke?
- How much do you feel you know about breast cancer?
- How is breast cancer discussed within families or communities?
- Do discussions change if someone is diagnosed?
- What sources of information about breast cancer do you rely on?
- Do you think breast cancer affects Black women differently? Why?

Prompts:

- Causes and risk factors
- Prevention and early detection
- Cultural beliefs
- Awareness within communities

**Domain 2: Opportunity — Social and Environmental Influences**

- Have you ever been invited for breast screening?
- What were your initial thoughts when you received the invitation?
- Were you aware of breast screening before receiving the invitation?
- What has been your experience with breast screening services?
- What factors influenced your decision to attend or not attend?
- Why do you think some Black women do not attend screening?
- Have you ever missed an appointment? Why?
- Do women born in the UK view screening differently from those born outside the UK?

Prompts:

- Appointment flexibility
- Work and caregiving responsibilities
- Healthcare experiences
- Cultural and religious influences
- Communication and invitation letters

**Domain 3: Motivation — Emotional Responses and Beliefs**

- What emotions are associated with breast screening?
- What concerns do women have about screening?
- What might encourage women to attend?
- How do family or peer experiences influence decisions?

Prompts:

- Fear of diagnosis
- Anticipated pain
- Body image concerns
- Trust in healthcare professionals
- Perceived benefits of early detection

**Domain 4: Suggestions for Improvement**

- How can access to breast screening be improved?
- What would make screening more acceptable for Black women?
- What changes to services would you recommend?
- Who should help raise awareness?

Prompts:

- Flexible appointments
- Community-based screening
- Media and awareness campaigns
- Role of GPs, community leaders, and family

**Closing**

- Final comments or additional thoughts
- Debrief and signposting to support services
- Thank participants and provide e-voucher information
